# Utilization of Primary Cytoreductive Surgery for Advanced-Stage Ovarian Cancer

**DOI:** 10.1001/jamanetworkopen.2024.39893

**Published:** 2024-10-16

**Authors:** Alexandra Bercow, Taylor Stewart, Amy J. Bregar, Allison Gockley, Varvara Mazina, J. Alejandro Rauh-Hain, Alexander Melamed

**Affiliations:** 1 Division of Surgery, Department of Gynecologic Oncology and Reproductive Medicine, University of Texas MD Anderson Cancer Center, Houston; 2 Meigs Division of Gynecologic Oncology, Vincent Department of Obstetrics and Gynecology, Massachusetts General Hospital, Boston

## Abstract

This cohort study examines changes in surgical approach for patients with stage III and IV epithelial ovarian cancer from 2010 to 2021.

## Introduction

Despite single-institution observational studies demonstrating a survival advantage with primary cytoreductive surgery (PCS) in advanced epithelial ovarian cancer (EOC),^1^ 4 randomized clinical trials have found that neoadjuvant chemotherapy (NACT) followed by interval cytoreductive surgery (ICS) achieves similar progression-free and overall survival, with decreased postoperative morbidity, as PCS.^2^ National guidelines, however, continue to recommend PCS as the preferred approach for patients with a high likelihood of achieving an optimal resection (<1 cm).^3^ Nonetheless, the utilization of NACT in the US has increased over time.^4^ In this study, we describe temporal and age trends in the up-front surgical treatment of patients with advanced EOC.

## Methods

In this retrospective cohort study, we included patients diagnosed with stage III to IV EOC from 2010 to 2021 in the National Cancer Database. We categorized patients as having received PCS, NACT followed by ICS, or no cytoreductive surgery. We fit Poisson regression models with robust standard errors to model temporal and age trends in the prevalence of treatment modalities and used these models for data visualization and to estimate rate ratios (RR) and 95% CIs. The study conformed to the Strengthening the Reporting of Observational Studies in Epidemiology (STROBE) guidelines.^5^ This study was deemed exempt from review and the requirement for informed consent by the Massachusetts General Hospital institutional review board because of the deidentified nature of the data. All analyses were performed with Stata statistical software, version 17.0 (StataCorp). Statistical significance was inferred when 95% CIs excluded the null.

## Results

**Figure 1.**
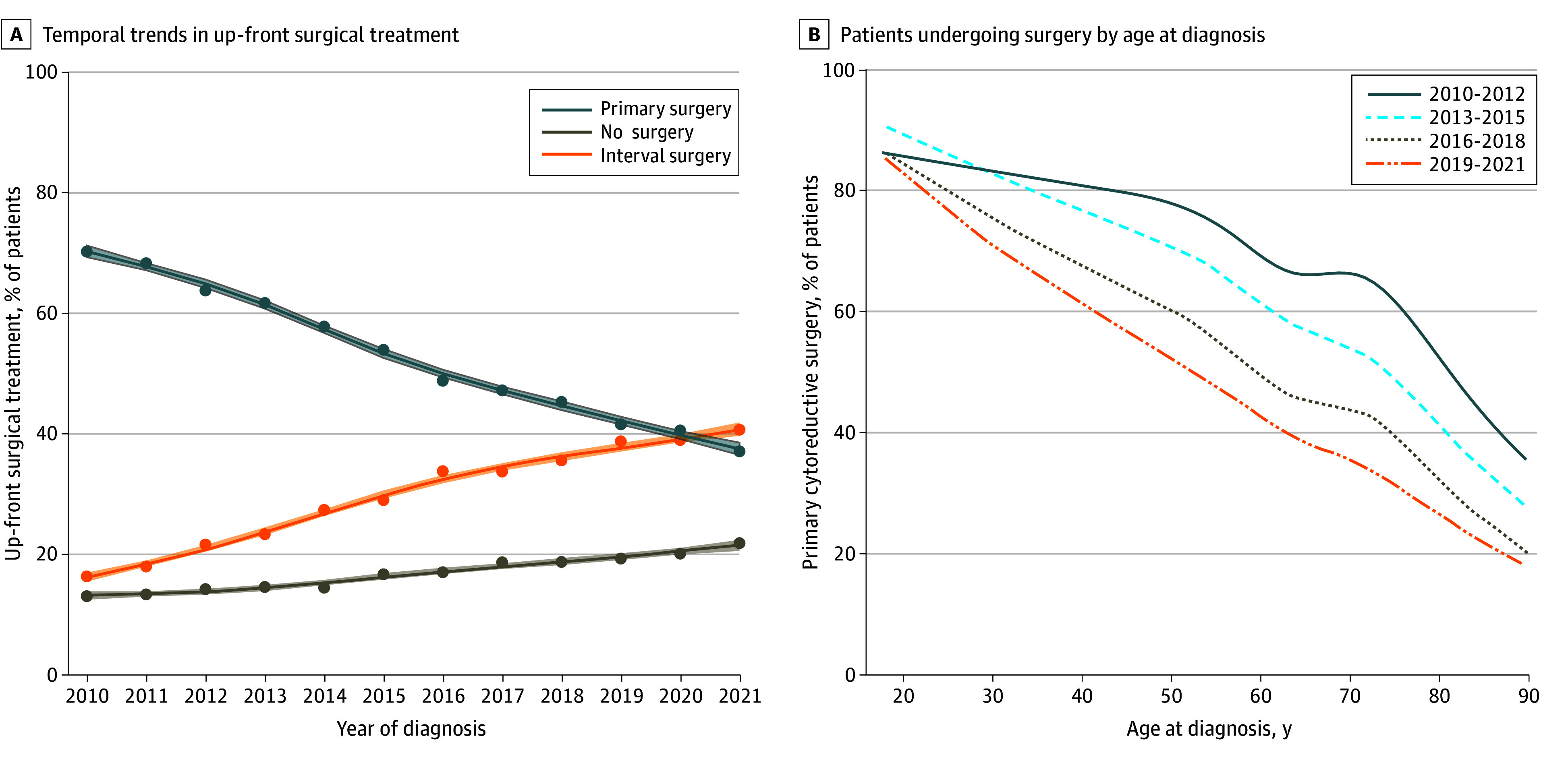
Temporal and Age-Related Trends in the Primary Treatment of Advanced Ovarian Cancer in the US

**Figure 2.**
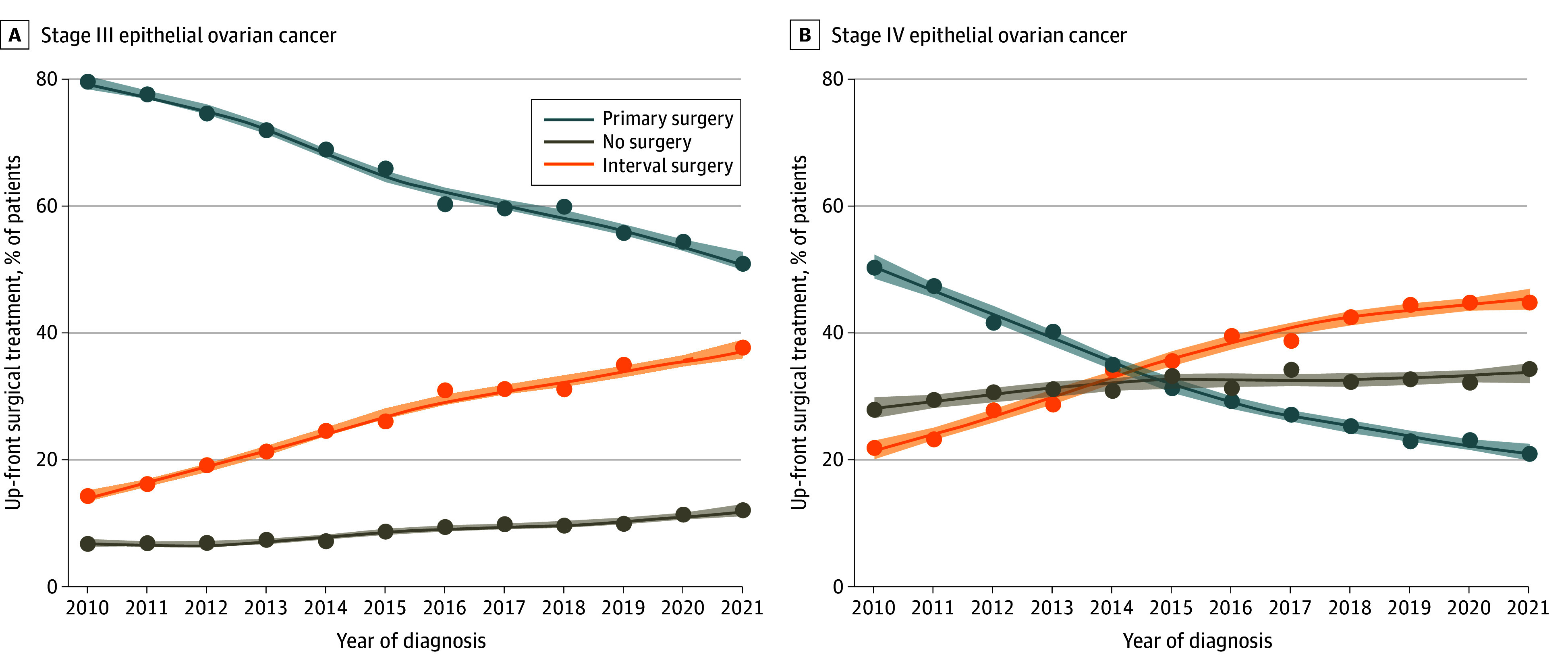
Temporal Trends by Stage in the Primary Treatment of Advanced Ovarian Cancer in the US

We identified 87 449 patients (mean [SD] age, 63.7 [12.4]), of whom 55 717 (63.7%) had stage III disease. Overall, 46 754 patients (53.5%) underwent PCS, 25 893 (29.6%) underwent ICS, and 14 802 (16.9%) received no surgery. The use of PCS and ICS changed dramatically over the study period, with ICS overtaking PCS as the most frequent treatment approach by 2021 (Figure 1A). From 2010 to 2021, the percentage of patients who underwent PCS fell from 70.1% to 37.2% (RR, 0.54; 95% CI, 0.52-0.55). The percentage of patients who underwent ICS increased from 16.6% to 40.8% over the same period (RR, 2.49; 95% CI, 2.36-2.61). There was a concurrent rise in the proportion of patients who received no surgery from 13.3% to 22.0% (RR, 1.62; 95% CI, 1.52-1.73). Substantial declines in the use of PCS were observed throughout the age ranges in which EOC is most frequently observed (Figure 1B). Among patients with stage III disease, the proportion of patients who underwent PCS fell from 79.3% to 51.1% (RR, 0.65; 95% CI, 0.63-0.67) compared with 50.2% to 21.0% (RR, 0.42; 95% CI, 0.39-0.45) among patients with stage IV disease (Figure 2A and B).

## Discussion

In the decade following the publication of the first randomized clinical trial that found NACT followed by ICS was noninferior to PCS for advanced-stage EOC, there has been a paradigm shift in the up-front treatment of patients with this disease in the United States. A substantial decline in the use of PCS occurred in conjunction with a large rise in the use of NACT and ICS and a modest increase in the proportion of patients who did not undergo any surgery. Since 2021, NACT followed by ICS has become the most prevalent approach for the up-front treatment of advanced EOC. While the trend was largely observed among those with stage IV disease, the use of NACT has increased significantly across all stages and ages. Historically, NACT was reserved for older patients, but our age-related findings demonstrate that the trend of increasing NACT use was not associated with one age group. The decline of PCS may be driven by recognition of the excess operative morbidity and mortality that results from this treatment approach.^6^ Additionally, the timing with which NACT overtook PCS as the major initial approach may be partially explained by the COVID-19 pandemic and initial limited operating room capabilities that resulted nationally.

## Notes

Supplement.
Data Sharing Statement
