# Error in Figure

**DOI:** 10.1001/jamanetworkopen.2024.56651

**Published:** 2024-12-19

In the Research Letter titled “Utilization of Primary Cytoreductive Surgery for Advanced-Stage Ovarian Cancer,”^1^ published October 16, 2024, there was an error in Figure 2. The left-hand panel should have been labeled “Stage III epithelial ovarian cancer,” and the right-hand panel should have been labeled “Stage IV epithelial ovarian cancer.” This article has been corrected.^1^
