# Supplementary material for: Utilization of Primary Cytoreductive Surgery for Advanced-Stage Ovarian Cancer
Source: JAMA Netw Open. 2024 Oct 16;7(10):e2439893. doi: 10.1001/jamanetworkopen.2024.39893 (PMC11581664; doi:10.1001/jamanetworkopen.2024.39893)
Supplement: Supplement. — Data Sharing Statement [file jamanetwopen-e2439893-s001.pdf]

Data Sharing Statement

Bercow. Utilization of Primary Cytoreductive Surgery for Advanced-Stage Ovarian Cancer in the US. *JAMA Netw Open*. Published October 16, 2024.
doi:10.1001/jamanetworkopen.2024.39893

Data
Data available: No

Additional Information
Explanation for why data not available: NCDB is widely available but we are not allowed to share this data per the NCDB data agreement.
